# Acoustic Sensor Design for Dark Matter Bubble Chamber Detectors

**DOI:** 10.3390/s16060860

**Published:** 2016-06-10

**Authors:** Ivan Felis, Juan Antonio Martínez-Mora, Miguel Ardid

**Affiliations:** Institut d’Investigació per a la Gestió Integrada de les Zones Costaneres (IGIC), Universitat Politècnica de València (UPV), 46730 Gandia, València, Spain; jmmora@fis.upv.es (J.A.M.-M.); mardid@fis.upv.es (M.A.)

**Keywords:** dark matter, bubble chamber, piezoelectric sensors, acoustic transducers, acoustic detection, acoustic test bench

## Abstract

Dark matter bubble chamber detectors use piezoelectric sensors in order to detect and discriminate the acoustic signals emitted by the bubbles grown within the superheated fluid from a nuclear recoil produced by a particle interaction. These sensors are attached to the outside walls of the vessel containing the fluid. The acoustic discrimination depends strongly on the properties of the sensor attached to the outer wall of the vessel that has to meet the requirements of radiopurity and size. With the aim of optimizing the sensor system, a test bench for the characterization of the sensors has been developed. The sensor response for different piezoelectric materials, geometries, matching layers, and backing layers have been measured and contrasted with FEM simulations and analytical models. The results of these studies lead us to have a design criterion for the construction of specific sensors for the next generation of dark matter bubble chamber detectors (250 L).

## 1. Introduction

Understanding the nature of dark matter is one of the most important goals in modern particle physics. A leading hypothesis to explain dark matter is its interpretation as a relic density of cold, non-baryonic Weakly Interacting Massive Particles (WIMPs). To check this hypothesis, direct detection dark matter experiments aim at observing the nucleus recoiling from the collision of a WIMP with ordinary matter. Different detector technologies have been proposed and used to this aim, being the superheated detector technology using refrigerant targets one of the most promising techniques due to its signficant advantage of being almost non-sensitive to electron recoils.

The PICO collaboration (formed from the merger of PICASSO and COUPP collaborations) uses bubble chamber detectors consisting of a fused-silica jar filled with hydraulic fluid and taped with a buffer layer. Several lead zirconate (PZT) piezoelectric acoustic transducers epoxied to the external wall of the fused-silica jar register the acoustic emission from bubble nucleations [1,2,3].

**Figure 1:**
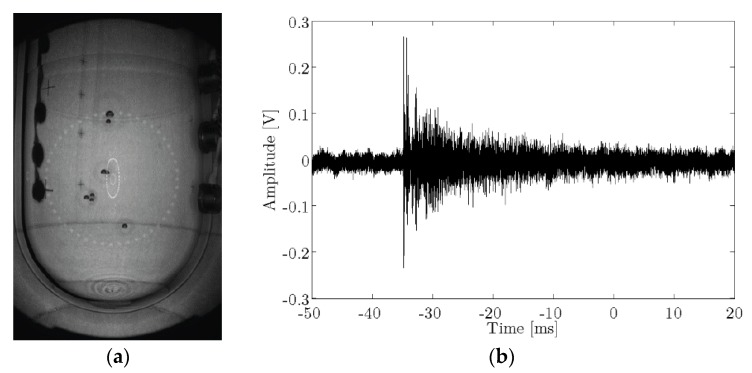
(**a**) image of PICO-60 during a multi-bubble neutron event due to multiple scattering inside the detector [2]; (**b**) typical acoustic signal recorded by a PICO acoustic sensor.

The Figure 1a shows an image of the PICO-60 acoustic sensors (developed by PICO collaboration, placed at SNOLAB underground laboratory, Sudbury, ON, Canada) mounted on vertical strings and epoxied to the external surface of the fused-silica jar. These sensors record the acoustic emission from bubble nucleation. A typical recorded acoustic bubble growth signal from a neutron calibration event is shown in Figure 1b. The sound of this bubble is quite impulsive and, therefore, broadband with frequencies up to 200 kHz. For the application in PICO, the signals are analyzed by means of an acoustic parameter that takes into account the acoustic power in different frequency bands corrected for the position of the bubble within the chamber. This parameter plays a crucial role in discriminating between different kinds of interacting particles [4] allowing to discriminate alpha radiation from the WIMP signal. This, together with the inherent rejection of the technique to beta, gamma, and muon radiation, and the possibility to discriminate neutrons through multi-bubble production will allow having large detectors with almost no background.

The developers of the acoustic system of these detectors have to keep in mind that, on one hand, the sensor system should be optimized for the transient (broadband) signal in a reverberant environment, involving several multilayers including the wall of the vessel; and, on the other hand, that there are serious constraints in the quantity of PZT material and on the reliability of systems in such detectors. In PICO detectors, radioclean salts with 100 times less radioactivity than usual ones are used, but even so, there are limitations in the total amount of PZT allowed in order to avoid “fake” events induced by radioactivity that will severely decrease the sensitivity of the experiment. Therefore, setting the best configuration for using the PZT available, either as disc or cylinder ceramics, is paramount. Reliability is another key issue since those sensors will be integrated in detectors that have to be in operation in deep underground labs, such as SNOLAB, for years. Once the system is, thus, defined and integrated it becomes almost impossible to intervene later in order to amend design mistakes or sensor failures. Within this frame, further research to improve the quality of acoustic sensors and of the analysis techniques needed will become more important for larger detectors, as for the next generation of PICO detectors (250 L). In this line, our group has built an acoustic test bench for R and D studies to optimize PICO-like acoustic sensors through all design steps. More specifically, our studies focus on knowing and improving the acoustic sensitivity of glued-to-wall sensor type in the frequency range up to 150 kHz. Studies on the characterization and simulation of piezoelectric materials, acoustic calibration and optimization of acoustic sensors are presented in this work. In this sense, as it will be discussed along the paper, this work used methods from previous research studies [5,6,7,8,9,10,11,12,13,14,15,16,17,18] in a multi-approach way to the problem: theory, simulation, and experiment, which help to better understand the situation and set the best conditions for multi-layer use in disc or cylinder piezoelectric transducer configurations for dark matter bubble chamber detectors.

The present paper is structured as follows. In Section 2, the determination of electromechanic coupling factors of different ceramic shapes is described. Several shapes of PZT based ceramics (PIC 255) were selected for our measurements. The correct implementation of this methodology will be tested in further radio-pure ceramics that have to be made *ad hoc* in radio-clean detectors. In Section 3, the numerical simulations of electric impedances in cylinders and discs with different diameter-to-thickness ratio (d/t) and the resulting deformation in several free modes are presented. From all simulated sizes, we selected two reference piezoelectric samples called “cylinder” and “disc” type ceramics, respectively, to better conduct and present our studies. In Section 4, the acoustic characterization of these selected samples is done by comparing the acoustic sensitivities obtained in a water tank with those obtained gluing the ceramics to a water filled glass vessel. In Section 5, increasingly complex designs are introduced and tested by the addition of different aluminium matching layer (ML) lengths ahead of the ceramic. This study was done both for the free ceramics and glued to the vessel ceramics. A multilayer analytical model was implemented in order to compare the measured increasing sensitivity peaks with those expected. In Section 6, the influence of adding an additional layer behind the ceramics (backing) is presented. Finally, the sensitivities in all configurations are compared and discussed extracting conclusions for multi-layer acoustic sensors, in general, and for the particular application in this kind of detectors.

## 2. Electromechanical Characterization

**Figure 2:**
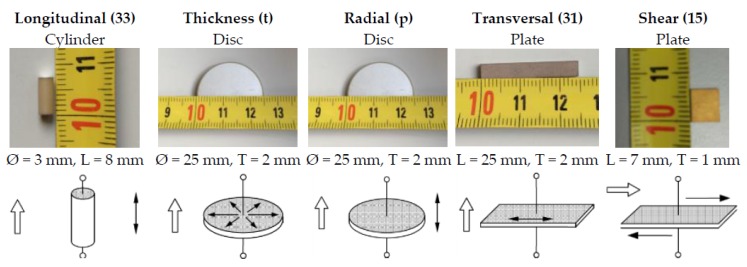
PIC255 ceramics used for obtaining piezoelectric coefficients following DIN EN 50324-02 standard. The hollow and solid arrows indicate the polarization direction and the displacement direction, respectively.

The determination of complete piezoelectric coefficients is performed based on the EN 50324-02 standard [6] in which ceramics with different polarizations and geometries are used. According to this standard, the resonance ($f_{r}$) and antiresonance ($f_{a}$) frequencies of five different geometry free-vibrating ceramics with the same piezoelectric material (in our case PIC255, PI Ceramic GmbH, Lindestraße, Germany) properly polarized (characterizing the five distinct modes of vibration, see Figure 2) are measured with an impedance analyzer. From each of them, the corresponding electromechanical coupling factors (k) are obtained. Additionally, by knowing the resonance and antiresonance frequencies, the measurements of the geometry (width, length, thickness, radius) and the material density each of the coefficients of the piezoelectric matrices [6] can be obtained. Figure 2 shows, on top, the ceramic shapes and sizes used in this study and, on the bottom, the main modes studied. From each of these samples, real and imaginary parts of the electrical impedance have also been measured with a Wayner Kerr Electronics 6500P impedance analyzer (London, England).

Once the full set of piezoelectric coefficients have been obtained experimentally, numerical simulation of the electrical impedance of each ceramic type can also be obtained. Thus, from simulated resonant and anti-resonant frequencies, the coupling factors k are obtained. Table 1 shows the results measured and obtained from simulations compared with those from the manufacturer datasheet. The results stand in agreement except in the case of the measured cylinder ($k_{33}$), probably due to holding effects in the experimental setup. The typical error for the resonance and/or anti-resonance frequency measurements of a specific sample in the production process is about 5% in fundamental modes, e.g., radial mode, and about 10% in coupled modes, e.g., thickness mode.

Good piezoelectric sensors can be produced, in principle, with a large coupling factor. The most used and common type of piezoelectric transducer elements in ultrasonic broadband applications is a thin piezoelectric shape with lateral dimensions much greater than thickness, driven in a simple thickness extensional mode of vibration [7]. However, as stated before, high-sensitivity transducers for a frequency range up to 150 kHz are needed in our application. Additionally, avoiding large ceramics for radioactivity limitations, we decided to study other modes with cylinders and discs that have lower frequency modes associated with longitudinal and radial modes, respectively.

## 3. Numerical Simulations

**Figure 3:**
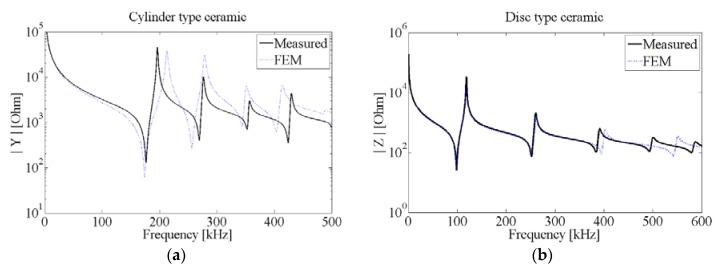
Impedance measurements and FEM simulations for PIC 255 cylinder (**a**) and disc (**b**).

The FEM simulations of piezoelectric ceramics were carried out in COMSOL Multiphysics. The input parameters are the coefficients of the elasticity matrix, the coupling matrix, the permittivity matrix, the density, and both mechanical and dielectric losses, respectively. The geometrical shape was set to match experimental conditions of tested ceramics with free boundary conditions and surface electrodes at electrical potentials and ground, respectively. Among the wide range of possible outputs that can be calculated in COMSOL, neither the electrical impedance nor the admittance can be obtained directly. However, we can calculate them from the inward surface charge density in the electrodes and the potential. The Figure 3 shows the comparison of the measurement results and the FEM simulations of the two PIC 255 ceramic transducers studied with 25 mm-diameter and 2 mm-thickness (disc), and 10 mm-diameter and 5 mm-thickness (cylinder) dimensions. In the experiment, the voltage was fixed at 1 V_pp_ and the frequency was swept from 100 Hz to 1 MHz with a step frequency of 100 Hz. This wide frequency range was chosen to see not only the first, but the several lowest modes. It can be observed that both results are in quite a good agreement, especially for low frequencies. Discrepancies on higher frequencies are due to some simplification on the geometry, not considering the electrode thickness, or due to mesh limitations [8]. Anyway, our study focused on the lowest frequency modes.

The local minima and maxima that appear in the impedance curve correspond to the resonance and anti-resonance peaks, respectively, of the radial modes (low frequency). With this pair of values we can calculate the generic electromechanical coupling factor $k=\sqrt{1-{\left(f_{r}/f_{a}\right)}^{2}}$ for the lower and higher modes using numerical simulations [8,9,10].

**Figure 4:**
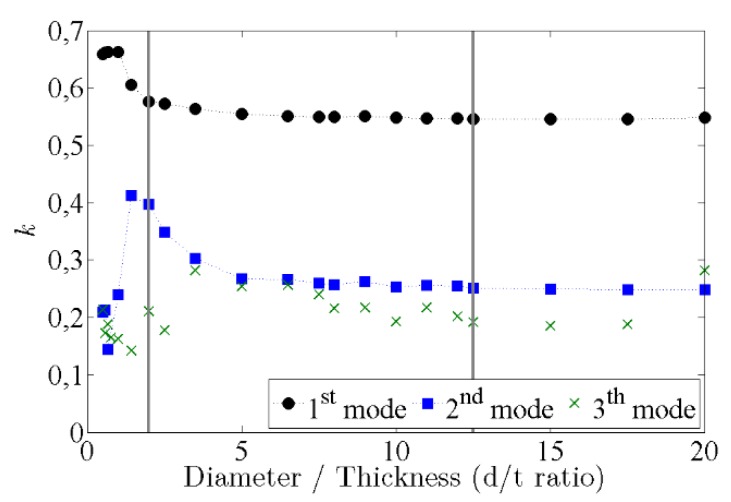
Results of the electromechanical coupling coefficients k from FEM simulations for the extensional vibration modes. The vertical lines indicate the selected ceramics for our studies.

Simulations have been made for several cylindrical and disc ceramic shapes, with different diameter-to-thickness ratios (d/t) between 0.5 (the most cylindrical shape) and 20 (the most disc-like shape) and the corresponding coupling factors k for the three lowest modes were calculated as shown in Figure 4. It can be seen that the best coupling for the lowest mode occurs when *d*/*t* < 1. On the other hand, there is a minimum in the second radial mode in this range, suggesting that there is a weak interaction between modes [8]. For intermediate values of the *d*/*t* ratio, between 1 and 10, a complicated vibrational spectrum appears, which increases the coupling of higher modes. For values of the ratio *d*/*t* larger than 10 the coupling factors of the lowest mode remains constants and those of higher modes decrease slightly. From the information of this plot, we have chosen and studied the acoustic behaviour and made improvements for two piezoelectric samples: a cylinder of 10 mm-radius and 5 mm-thickness (*d*/*t* = 2) and a disc of 25 mm-radius and 2 mm-thickness (*d*/*t* = 12.5), indicated in vertical lines in Figure 4. These geometries are similar to the ceramics already used in PICO-2L and PICO-60 detectors. With these two geometries, we can compare the acoustic behaviour of a ceramic sensor with and without high mode coupling, as explained in the following sections.

**Figure 5:**
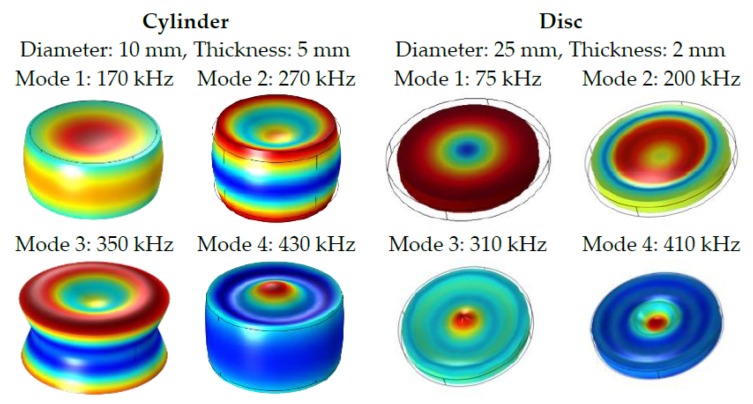
Admittance anti-resonance frequencies of the first four lowest modes for the reference cylinder and disc samples.

It is also interesting to observe the different dynamical vibration of both selected geometries calculated from FEM simulations as shown in Figure 5. The first mode in the disc is an almost pure radial vibration, with a large radial compression and elongation and a weak thickness deformation. As the mode is increased, a larger thickness deformation appears with different maxima and minima elongations along the radius. The deformations in the cylinders are not so clear. The first mode is a merger of radial and thickness deformation with minimum elongation in the edges and a maximum deformation in the center plane (radial component) and along the longitudinal axis (thickness component). In the higher modes the deformations are more evident with more maxima and minima along the radius.

As explained, in many applications of these acoustic sensors, and more specifically in bubble chamber detectors, the received signal is a bandwidth pulse (considered here up to 150 kHz) and the ceramics are attached to the external wall of a superheated liquid filled vessel. Thus, the acoustic signal covers only the first mode in this cylinder and the two first modes in the disc. The behavior of these modes can be affected by the way it is attached in the sense that one of the planar surfaces has more rigidity, *i.e.*, less freedom to vibrate. All of these aspects can affect the acoustic sensitivity of the final transducers. In addition, the acoustic sensitivity will be modified by inserting a new layer in the middle of the wall and the piezoelectric ceramic.

## 4. Acoustical Characterization

### 4.1. Calibration Setups

The sensitivity of each transducer has been quantified by the so-called received voltage response (RVR). Due to the spatial limitation of the measurement system and to the frequency range considered in the application, the sensitivity was calculated from 30 kHz to 150 kHz.

**Figure 6:**
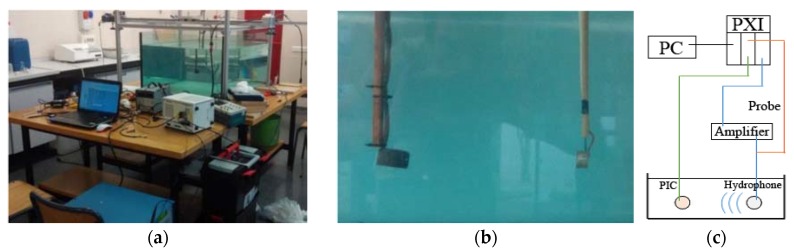
Configuration 1: measurements in the water tank. (**a**) The experimental setup; (**b**) image of the calibration of a ceramic with an FFR SX30 reference hydrophone; (**c**) scheme of the experimental setup.

**Figure 7:**
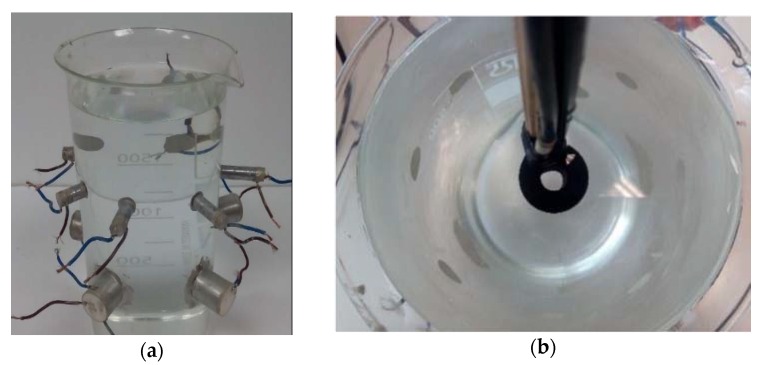
Configuration 2: measurements in a testing vessel. (**a**) Image of the vessel with all ceramics attached; (**b**) calibration with an FFR SX30 reference transducer. The scheme of the experimental setup is the same than in configuration 1.

The ceramics were measured in two configurations that can be seen in Figure 6 (configuration 1: free ceramics) and Figure 7 (configuration 2: glued ceramics). In configuration 1, the transducers are free inside of a water tank and, in configuration 2, they are attached to a water-filled 11.5 cm-diameter and 2.2 mm-thickness glass vessel. All measurements are controlled by the generation-reception NI-PXI-1031 system in LabView (National Instruments Corporation, Austin, TX, USA) and the signals are post-processed in MATLAB (The MathWorks, Inc. Natick, MA, USA). The experimental method and the signal processing techniques are similar to the ones presented in [5,12].

Regarding configuration 2, we have to take into account that there is a closer distance between the emitter and receiver. With this configuration it is difficult to reach a free acoustic field for the whole frequency range studied. Thus, the acoustic signal can be transmitted to the glass wall and some reflections can arrive to each transducer before the end of the direct reception of the acoustic signal. In order to discriminate between direct and reflection waves, we used cross-correlation and discrimination methods, which were successfully tested before [12]. These difficulties are conveniently avoided in the water tank calibration (configuration 1). However, these aspects also appear in the PICO-2L and PICO-60 bubble chambers, being an intrinsic issue of acoustics in bubble chambers.

### 4.2. Measurements of the Free and Glued Ceramics

**Figure 8:**
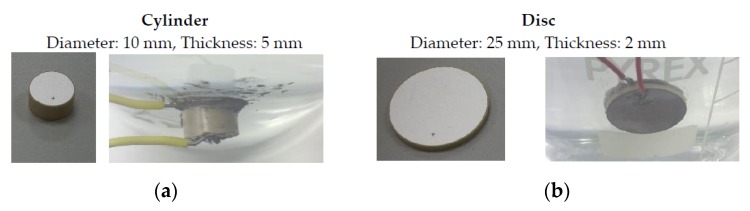
Pictures of the reference cylindrical (**a**) and disc (**b**) ceramics, free and attached to the vessel.

Figure 8 shows the cylindrical (left) and disc (right) ceramics used in our experimental setup, both free and directly glued to the vessel. The attaching glue is a conductor epoxy CW2400 (Chemtronics, Kennesaw, GA, USA).

**Figure 9:**
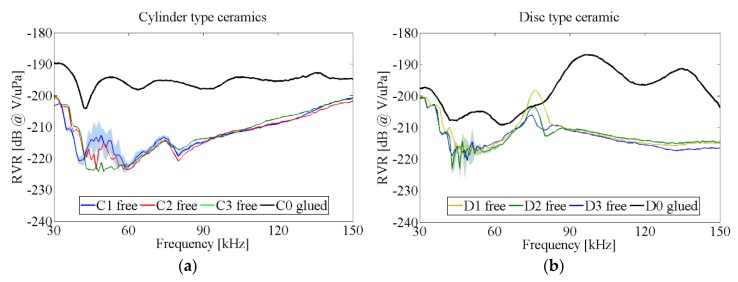
RVR of ceramics in configurations 1 (free) and 2 (glued). As an example, the shadowed area shows the uncertainties for a ceramic of each type, being quite similar for the rest of ceramics. The uncertainties are small for frequencies above 60 kHz, less than 1 dB, but it may be larger, up to 5 dB, for frequencies below 60 kHz. (**a**) Cylinder type ceramic; (**b**) Disc type ceramic.

Figure 9 shows the RVR, *i.e.*, the sensitivity of each ceramic type (cylinder and disc), both free in the water tank (configuration 1) and the corresponding one glued to the vessel (configuration 2).

With respect to the configuration 1, it can be seen that the maximum of sensitivity corresponds to the frequency location of the maximum admittance, about 80 kHz, for both cylinder- and disc-type ceramics. In addition, the sensitivity peaks in the disc case are narrower (a few kHz) than in the cylinders (tens of kHz). Thus, larger sensitivities at low frequencies can be reached with free discs, but larger bandwidths in free cylinders. However, this behavior changed when the ceramics were glued to the vessel. With respect to configuration 2, it can be seen that, in general, placing both ceramics in the vessel increases the sensitivity due to a better impedance matching (water-vessel-ceramic) than for the free ceramic case (water-ceramic). This is an intrinsic acoustic gain for the characteristic setup in bubble chambers. The cylindrical ceramic has a larger and flatter sensitivity than the disc one. In both cases, the maximum sensitivity peak appears approximately at the same frequency. This suggests that the vessel wall has little effect on the behavior of the lowest piezoelectric modes. In other words, the rigidity of the Pyrex glass wall was not sufficient to completely constrain the ceramic deformation.

From Figure 9 we can conclude that relatively high sensitivity sensors (RVR = −195 ± 3 dB @ V/μPa) can be obtained by using the cylindrical piezoceramics in the full frequency range (from 30 kHz to 150 kHz), and also with the disc ceramic, but only in the frequency range from 80 kHz to 140 kHz. In order to improve the acoustic response of the final transducer design the optimization of matching layer and backing is discussed in next sections.

## 5. Optimization of the Matching Layer

As discussed, the acoustic sensors of PICO detectors are glued on the outer walls of the vessel containing the target fluid. Therefore, the design should be optimized for a good acoustic transmission between the fluid and the piezoceramic, with the restriction that there is an intermediate layer (quartz wall) which cannot be, in principle, modified. A matching layer can be used to improve transmission. For the selection of the material, as first approximation, knowing the characteristic acoustic impedance of the quartz or Pyrex ($Z_{i}\;\sim \;11.0\;\;\text{MRayls}$) and of the piezo ($Z_{t}\;\sim \;18.4\;\;\text{MRayls}$), the ideal matching layer (ML) between them should have an impedance of $Z_{1}=\sqrt{Z_{i}\cdot Z_{t}}=14.2\;\text{MRayls}$ [7]. In this first study to evaluate the multilayer theoretical model with experimental results, we have chosen the aluminum as ML. Despite its acoustic impedance ($Z_{1}=17.2\;\text{MRayls}$) not being the best, it has a rather good impedance and it is affordable, with low attenuation for acoustic waves and easy to machine, so different thickness could easily be compared. We implemented a multilayer acoustic transmission model and made several experimental measurements to test it with the aim at taking a proper decision on the ML length and material.

### 5.1. Characterization of Free Ceramics with Matching Layer

**Figure 10:**
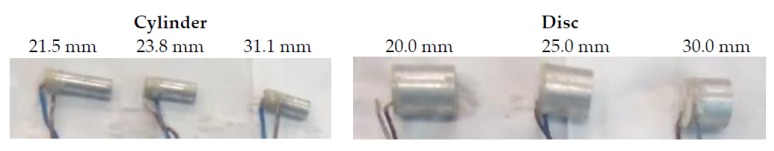
Aluminum matching layers attached to the free ceramics.

The sensitivity of cylinder and disc ceramics with different aluminum ML lengths were measured in the water tank, as can be seen in Figure 10. They were glued to the ceramic with the conductive epoxy CW2400 pressing with a mechanical jack in order to have a good reproducibility in the bonding conditions. The aluminum lengths were selected in order to increase the sensitivity (maximize the water-glass-ML-piezo transmission) in the 40–70 kHz frequency range.

**Figure 11:**
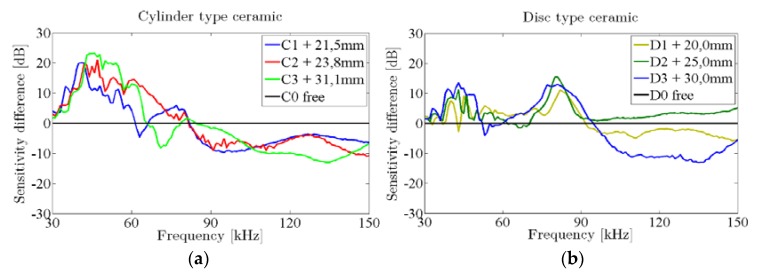
Sensitivity difference of ceramics with ML (color lines) with respect to the one without ML (black line), measured in the water tank. The statistical uncertainties are quite similar to those shown in Figure 9. The estimated systematic uncertainty due to reproducibility is 3 dB. (**a**) Cylinder type ceramic; (**b**) Disc type ceramic.

Figure 11 shows the sensitivity difference between the ceramics with ML and the free one. For cylinders, it can be seen that the best efficiency is for 40 kHz approximately (the larger the ML length, the lower the frequency) with an increase of about 20 dB. Then, the sensitivity decreases, and it is smaller than for the free ceramic in frequencies higher than 80 kHz. In the case of discs, a sensitivity peak also appears at 40 kHz in all ML length but with an inferior gain (12 dB), and a second one can be seen around 80 kHz with similar increase. For higher frequencies, the sensitivity falls and only the 25 mm ML disc remains above (up to 3 dB) with respect to the free one. However, considering the systematic uncertainties, this result should be taken with caution.

### 5.2. Characterization of Glued Ceramics with Matching Layer

**Figure 12:**
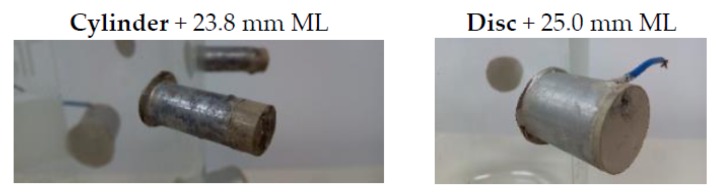
Aluminum matching layer attached to the glued ceramics for cylinders and discs.

Once the behavior of different ML in both ceramic types is known, they were glued to the vessel glass and their sensitivities measured. Figure 12 shows some pictures of these ceramics.

**Figure 13:**
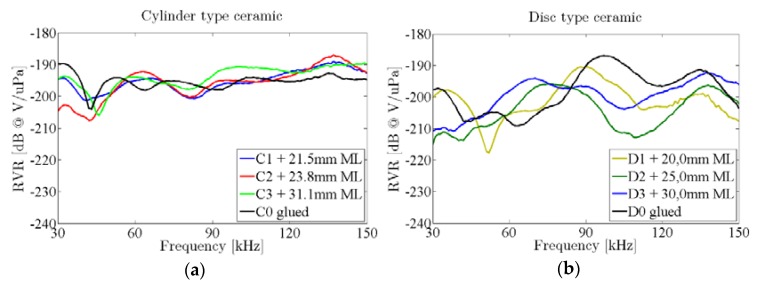
RVR of ceramics with ML (color lines) and without it (black line), glued to the vessel. (**a**) Cylinder type ceramic; (**b**) Disc type ceramic.

Figure 13 shows the RVR of the ceramics attached to the vessel. It can be seen that the sensitivity for discs with ML increases in the 50–70 kHz range with respect to the one without ML. As expected, the increase is larger for longer ML. The ML did not have any significant effect in the second resonance. The different behavior can be understood in terms that the ML lengths have been set to maximize the acoustic transmission in the system water-glass-ML-piezo for the frequencies of the lowest free modes. On the other hand, there is no clear increase of sensitivity in the cylinder ceramics with ML that were studied.

**Figure 14:**
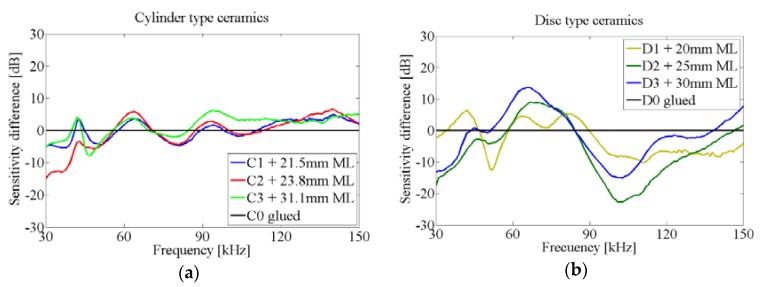
Difference of sensitivity in ceramics glued to the vessel, with and without ML. (**a**) Cylinder type ceramic; (**b**) Disc type ceramic.

Figure 14 shows the effect of adding a ML between the glass wall and the ceramic by considering the difference between the testing ceramics attached to the vessel with and without ML. There are many narrow peaks of increased sensitivity for the cylinder ceramics with ML, but usually the effect of the ML is small. However, the variations of sensitivity in discs due to ML can be large. For instance, there is a remarkable increase at 70 kHz and a big decrease in sensitivity for frequencies above 90 kHz. This effect can be understood in terms of the length of the sensors (ceramics + ML) which is now larger and there is now a higher rigidity. All of this is against the vibration in the lowest free modes, resulting in a large decrease in the RVR at these frequencies.

### 5.3. Comparison with Theoretical Models

With the aim at taking a proper decision of the ML length to be used for the sensors, we implemented a multilayer acoustic transmission model and tested it with the experimental measurements mentioned *supra*. The model consists of incorporating one or more acoustic layers between the incidence acoustic load and the receiving side of the piezoelectric element. Imposing the conditions of pressure continuity and particle velocity continuity at each of the resulting interfaces, the total acoustic reflection and transmission coefficient can be obtained. Comparing the transmission coefficient with and without the intermediate layers (ML), we can estimate their effect in the ceramic sensitivity. More detailed information in these models can be found in [13,14,15].

**Figure 15:**
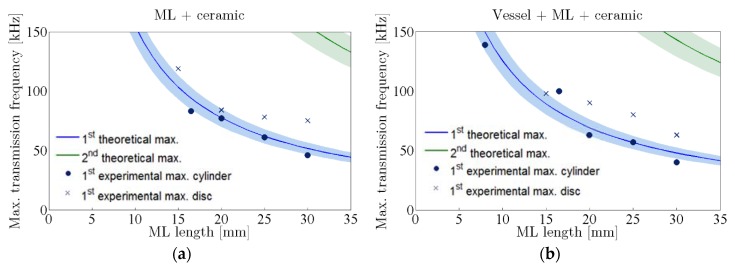
Frequency of maximum sound transmission *vs.* thickness of the aluminum ML for the models of transmission for one layer (**a**) and two layers (**b**), compared to the measured data in configurations 1 and 2, respectively.

For the implementation of the model we have considered that there is a first layer of 2.2 mm-thick Pyrex glass, which cannot be changed, and a second aluminum layer whose length can be varied arbitrarily. The frequency position of the measured first maximum sensitivity peaks in configurations 1 and 2 with different ML lengths are compared to the theoretical model for transmission through one and two layers, respectively. In this sense, Figure 15 shows the predicted theoretical maximum transmission frequency as a function of the ML length for both configurations. The color lines consider the average values of material properties and the shadowed bands takes into account their uncertainties. In addition, the frequency of the sensitivity peaks from Figure 10 and Figure 12 for each ML length and configuration are drawn. The circles (●) correspond to the case of cylinder-type sensors and the crosses (×) to the disc-type sensors. It can be seen that the expected frequency for the maximum sensitivity frequency in experimental setups fits quite well into the first theoretical peak (blue), especially in the case of cylinders because the transmission behaves more similarly to a plane wave, which is a condition that was applied in the model.

As a conclusion of this section, it can be stated that an increase in sensitivity can be obtained for a desired low frequency just by adding an intermediate ML to the piezoelectric ceramic. This improvement can reach up to 20 dB in the case of cylinders, and 12 dB in discs. However, when the ceramics with ML are glued to the vessel (two ML model), a small increase or decrease in sensitivity is observed for the cylinders depending on the frequency whereas, for the disc, there is a large increase (or decrease) in sensitivity for frequencies below (or above) 90 kHz, respectively. 

We have not considered the thickness of the glue between the glass wall and the ML in this theoretical model. Keeping this in mind, after testing this model, we studied the possibility to mismatch the acoustic impedance between the piezoceramic and the load by using two matching layers where one of them is the vessel and the other is the proper length of glue. Some studies can be found on this topic in [15].

## 6. Optimization of the Backing

Usually, a high backing attenuating material (BK) is bonded to the back side of the piezo transducer element in order to enlarge the reception frequency band and, therefore, to shorten the impulse response (at the expense of a loss in sensitivity and signal amplitude) [7]. There are several design considerations which need to be considered to select an appropriate backing layer [16]. On one hand, the impedance of the backing material has to be chosen according to the required bandwidth of the transducer. However, increasing the bandwidth by increasing the backing impedance also decreases the efficiency and, thus, the sensitivity [17]. This means that there is a tradeoff between these parameters. On the other hand, the attenuation coefficient of the backing material should be as high as possible so that acoustic waves transmitted backwards cannot be reflected back avoiding interference echoes. In addition, most typical models of backing assume that the thickness of the backing layer is semi-infinite and without losses [18]. Thus, the backing length has to be considerably thicker than the ceramic thickness.

In order to test the effect of an additional layer on the back of the tested transducers, several samples of EPO 4030 were made and tested glued to free disc ceramics. After that, we attached some of these samples to the ones glued to the vessel for the final transducer design consideration.

### 6.1. Characterization of Free Ceramics with Backing

**Figure 16:**
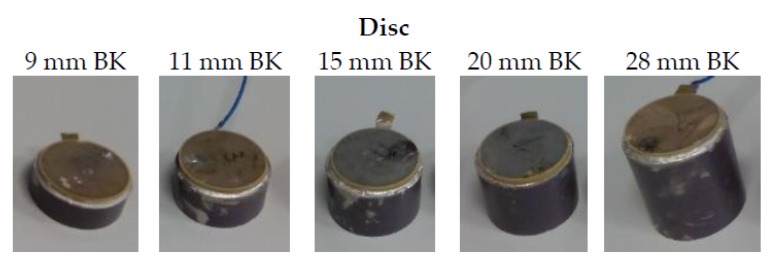
Backing layers attached to the disc ceramics.

Figure 16 shows pictures of the ceramics with the backing whose sensitivity have been measured in the water tank (configuration 1, Figure 7). The lengths of backing have been selected so that it is longer than several wavelengths and thicker than the ceramic.

**Figure 17:**
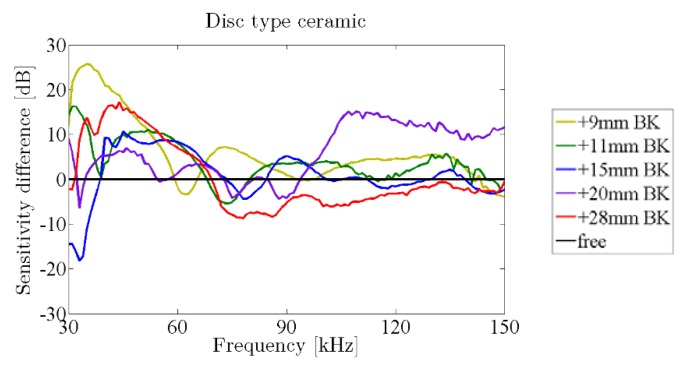
Sensitivity difference of ceramics with backing (color lines) with respect to the one without backing (black line) measured in the water tank.

Figure 17 shows the sensitivity difference of the free ceramics with backing with respect to the corresponding ones without backing. It can be seen that there is a good response in almost all cases studied up to 60 kHz, with the exception of the case of the 20 mm backing, in which a great sensitivity improvement is observed for frequencies above 100 kHz. Again, this response is modified when gluing the ceramics to the vessel.

### 6.2. Characterization of Glued Ceramics with Matching and Backing

**Figure 18:**
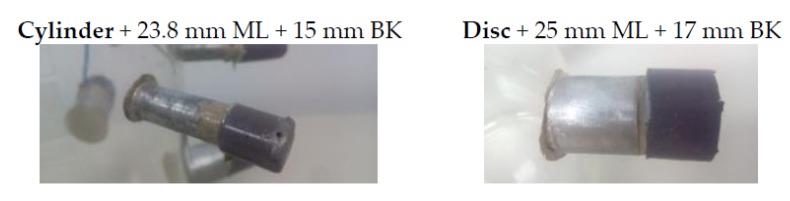
Glued ceramics with matching layer and backing.

After measuring the influence of a backing layer behind a free disc ceramic, we studied the acoustic response of the several backings in the already glued ceramics. Figure 18 shows a cylinder and a disc ceramic type with matching layer and backing glued to the vessel.

**Figure 19:**
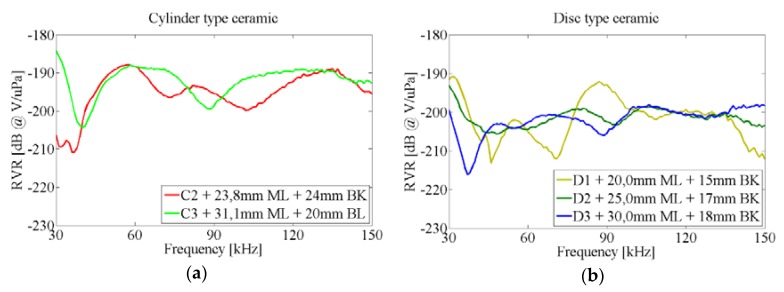
RVR of ceramics with backing and matching layer glued to the vessel. (**a**) Cylinder type ceramic; (**b**) Disc type ceramic.

Figure 19 shows the RVR of the transducers with matching layer and backing attached to the vessel. In the case of cylinders, the obtained sensitivities are quite flat, around −190 dB, with some decrease at lower frequencies. In the case of discs, the sensitivity is also flat, around −200 dB, but with some increase at lower frequencies. The ceramic with a shorter backing presents a more fluctuating behavior, but this ceramic already presented some different behavior when attached to the vessel with ML and without backing, so it is not possible to derive a solid conclusion about the effect of the backing.

**Figure 20:**
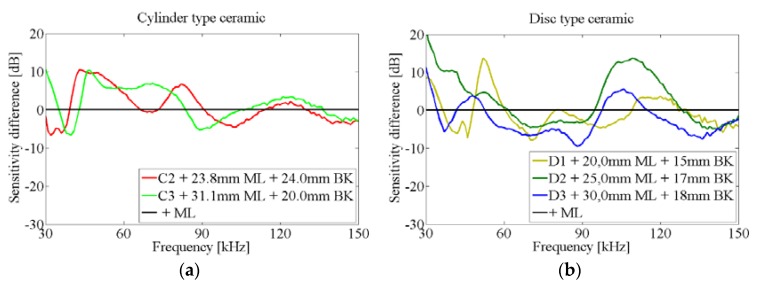
Difference of sensitivity in ceramics glued to the vessel, with and without backing. (**a**) Cylinder type ceramic; (**b**) Disc type ceramic.

The above behavior can be also seen comparing the sensitivity difference between each of the testing ceramics attached to the vessel with and without the backing, plotted in Figure 20. The differences in sensitivity are similar for both cylinder ceramics. For the discs, as in the case of free ceramics with backing, the acoustic improvement appears for frequencies below 60 kHz and above 100 kHz, with a decrease in sensitivity in between. However, it seems that the best improvements can be reached with the 17 mm BK. As expected, the addition of the backing enlarges the frequency width of the increased sensitivity peaks with respect to using the ML only.

## 7. Global Results and Conclusions

This study investigates the acoustic behavior of piezoceramic disks and cylinders in order to be used in dark matter bubble chamber detectors. For the desired features of those sensors, it must be underlined that they are attached to the outside wall of a glass vessel, that there are limitations in PZT amount and radiopurity, and that a good frequency bandwidth is required in correspondence with the acoustics of bubble growth in a superheated fluid in a reverberant environment. For the optimization of this kind of sensor, each design step had to be studied properly, taking into account some numerical simulations, analytical models, and experimental measurements.

The initial material characterization, discussed in Section 2, allowed us to reject the geometries associated with a low electro-mechanical coupling factor. In addition, with the complete set of piezoelectric coefficients of any piezoceramic material, we estimated the coupling modes and observed the mechanical deformation through numerical simulations, as seen in Section 3. Once glued to the vessel, these modes are modified and specific studies are required.

**Figure 21:**
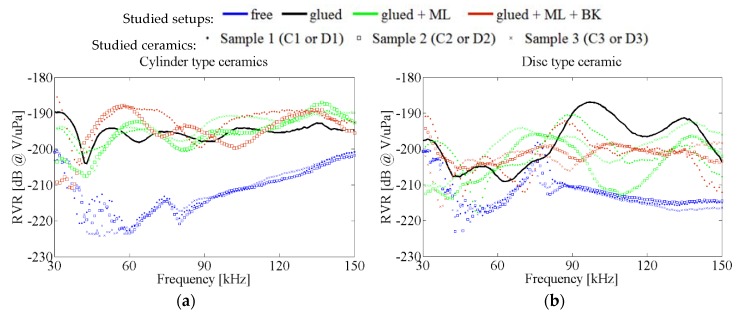
Acoustic sensitivity of all measured ceramics, with and without ML and BK. (**a**) Cylinder type ceramic; (**b**) Disc type ceramic.

The acoustic sensitivities of the samples were first measured in a water tank and, later on, glued to a test vessel, as explained in Section 4 and following paragraphs. Successively, free ceramics, ceramics with a matching layer, and ceramics with both a matching layer and backing were measured in those setups. Figure 21 summarizes the results shown in previous sections for the case of glued ceramics. The blue dots indicate the sensitivity of free ceramics in water tank, whose peaks corresponds to those seen in numerical deformations. The black line corresponds to a ceramic glued to the vessel. The increase in sensitivity obtained in this initial step is due to the fact that there is a better acoustic impedance transmission between water-glass-piezo than in free ceramics (water-piezo). Generally, the cylinders have a flatter response than discs with similar mean sensitivity. However, when we add an additional aluminum matching layer between the glass wall and the ceramic some peaks of increased sensitivity appears depending on the ML length (as expected analytically), but the sensitivity decreases in other frequency ranges, as well. For the disc type ceramics, flatter sensitivity transducers can also be chosen by adding an additional layer behind each transducer (backing). With this configurations, different transducers present similar sensitivities, achieving a good sensitivity with an observable flat response in frequency.

From the large variety of transducers analyzed, it is possible to select some of them that have a final desirable frequency response for the application proposed. Generally, cylinders have better sensitivity than discs. That leads to the selection of the cylinder with 31 mm ML and 20 mm BK as the most sensitive transducer with −190 dB @ 1 V/μPa. Another interesting solution is the disc with 25 mm ML and 17 mm BK since it presents the flattest response with −200 dB @ 1 V/μPa with high sensitivity at lowest frequencies as well.

Although the goal of this research was to be a first approach to the study of the behavior of ceramics in dark matter bubble chambers, we think that it is possible to extract important and general conclusions from the studies and results described: it is possible to understand the response of the ceramics from the analytical models and simulations and, as shown, this response can be adequately tuned through the addition of a matching layer and backing. The multi-layer model was quite useful to understand the response and to design the sensors. The different contributions could also be evaluated experimentally by using the two setups described. The utility of this process was demonstrated for both cylinder and disc ceramics.

## Figures and Tables

**Table 1:** Comparison of the PIC 255 electromechanical coupling factors (k) from the manufacturer datasheet, experimental measurements, and numerical simulations.

	$k_{33}$	$k_{p}$	$k_{t}$	$k_{31}$	$k_{15}$
Manufacturer	0.691	0.620	0.471	0.351	0.661
Measured	0.242	0.624	0.471	0.351	0.628
Simulated	0.695	0.619	0.564	0.346	0.689
